# Neuroprotective Effects of Nicotinamide against MPTP-Induced Parkinson’s Disease in Mice: Impact on Oxidative Stress, Neuroinflammation, Nrf2/HO-1 and TLR4 Signaling Pathways

**DOI:** 10.3390/biomedicines10112929

**Published:** 2022-11-14

**Authors:** Inayat Ur Rehman, Amjad Khan, Riaz Ahmad, Kyonghwan Choe, Hyun Young Park, Hyeon Jin Lee, Abubakar Atiq, Jungsung Park, Jong Ryeal Hahm, Myeong Ok Kim

**Affiliations:** 1 Division of Life Sciences and Applied Life Science (BK 21 Four), College of Natural Science, Gyeongsang National University, Jinju 52828, Republic of Korea; 2 Department of Psychiatry and Neuropsychology, School for Mental Health and Neuroscience (MHeNs), Maastricht University, 6229 ER Maastricht, The Netherlands; 3 Department of Pediatrics, Maastricht University Medical Center, 6202 AZ Maastricht, The Netherlands; 4 Division of Endocrinology and Metabolism, Department of Internal Medicine, Gyeongsang National University Hospital and Institute of Health Sciences and Department of Internal Medicine, College of Medicine, Gyeongsang National University, Jinju 52828, Republic of Korea; 5 Alz-Dementia Korea Co., Jinju 52828, Republic of Korea

**Keywords:** nicotinamide (NAM), 1-methyl-4-phenyl-1,2,3,6-tetrahydropyridine (MPTP), intraperitoneal (i.p), substantia nigra pars compacta (SNpc)

## Abstract

Nicotinamide (NAM) is the amide form of niacin and an important precursor of nicotinamide adenine dinucleotide (NAD), which is needed for energy metabolism and cellular functions. Additionally, it has shown neuroprotective properties in several neurodegenerative diseases. Herein, we sought to investigate the potential protective mechanisms of NAM in an intraperitoneal (i.p) 1-methyl-4-phenyl-1,2,3,6-tetrahydropyridine (MPTP)-induced Parkinson’s disease (PD) mouse model (wild-type mice (C57BL/6N), eight weeks old, average body weight 25–30 g). The study had four groups (*n* = 10 per group): control, MPTP (30 mg/kg i.p. for 5 days), MPTP treated with NAM (500 mg/kg, i.p for 10 days) and control treated with NAM. Our study showed that MPTP increased the expression of α-synuclein 2.5-fold, decreased tyrosine hydroxylase (TH) 0.5-fold and dopamine transporters (DAT) levels up to 0.5-fold in the striatum and substantia nigra pars compacta (SNpc), and impaired motor function. However, NAM treatment significantly reversed these PD-like pathologies. Furthermore, NAM treatment reduced oxidative stress by increasing the expression of nuclear factor erythroid 2-related factor 2 (Nrf2) and heme oxygenase-1 (HO-1) between 0.5- and 1.0-fold. Lastly, NAM treatment regulated neuroinflammation by reducing Toll-like receptor 4 (TLR-4), phosphorylated nuclear factor-κB, tumor (p-NFκB), and cyclooxygenase-2 (COX-2) levels by 0.5- to 2-fold in the PD mouse brain. Overall, these findings suggest that NAM exhibits neuroprotective properties and may be an effective therapeutic agent for PD.

## 1. Introduction

Parkinson’s disease (PD), a chronic and progressive neurodegenerative disease, is characterized by several motor dysfunction and non-motor dysfunctions including autonomic disturbances, behavioral and cognitive features [1]. Primarily PD is associated with the degeneration of dopaminergic neurons in the substantia nigra of the midbrain and the loss of their axonal project to the striatum, resulting in the loss of dopamine neurotransmitters and abnormal accumulation of α-synuclein in the brain [2]. Besides dopaminergic neuronal loss, oxidative stress and neuroinflammation are also key players in PD pathology [3].

Consequently, the symptoms are initiated, including rest tremors, bradykinesia, postural instability, and rigidity [4,5].

The pathophysiology of PD is heterogeneous and needs a diverse range of animal models that can describe different aspects of the disease.

1-methyl-4-phenyl-1,2,3,6-tetrahydropyridine (MPTP) is a widely used in vivo model to study PD. MPTP is a potent toxicant which damages dopaminergic neurons and produces α-synuclein in the striatum and the substantia nigra pars compacta (SNpc) [6,7]. Additionally, MPTP causes oxidative stress, neuroinflammation, neuronal cell death, and neurodegeneration. In the dopaminergic neurodegeneration, decrease activity of tyrosine hydroxylase (TH), and dopamine transporters (DAT) was also reported [8]. Along with these complications, oxidative stress produced by the accumulation of α-synuclein in the brain is the most dominant consequence of PD. The antioxidant defense markers, nuclear factor erythroid 2-related factor 2 (Nrf2) and heme-oxygenase (HO-1), are also disrupted in the brain [9]. Inflammation also plays a vital role in dopaminergic neurodegeneration. Glial cells are the resident macrophage of brain, which maintain the homeostasis by doing first line defense against various toxins. Activated glial cells also reported in MPTP-induced PD [10]. Further, activated glial cells trigger the inflammatory cascade in the brain by stimulating, Toll-like receptors (TLRs), which further release proinflammatory factors such as phosphorylated nuclear factor-κB, tumor (p-NFκB), and cyclooxygenase-2 (COX-2) [11,12].

Vitamins are an important dietary supplement and lacking or excess amount produced several diseases [13]. Nicotinamide (NAM) is a biologically active amide of vitamin B3 and is a precursor to the cofactor nicotinamide adenine dinucleotide (NAD), which is required for energy metabolism and other vital cellular activities [14]. NAM has demonstrated several beneficial effects and is used for several diseases. NAM showed potent anti-inflammatory properties against acne vulgaris [15]. NAM used as chemo preventive agent against skin cancer, showed effective treatment for autoimmune conditions, metabolic diseases [16], and skin aging [17]. NAM also provides protection for neurons against free radical injury, ethanol-induced neuronal injury, and protects against amyloid beta (Aβ) toxicity in Alzheimer’s disease and other neurological disorders [18,19,20]. Furthermore, the neuroprotective effects of NAM against apoptotic cell death were also investigated in an MPTP-induced PD mice model [21]. Therefore, in the present study, we investigated the neuroprotective potentials of nicotinamide in an MPTP mice model, and hypothesized that nicotinamide may exert neuroprotection on dopaminergic neurons against MPTP-induced oxidative stress, neuroinflammation, motor dysfunction, and neurodegeneration.

## 2. Materials and Methods

### 2.1. Chemicals and Antibodies

MPTP was obtained from Sigma-Aldrich (St. Louis, MO, USA), and 30 mg/kg was dissolved in 0.9% of sterile saline solution with high speed agitation [22,23] through proper laboratory safety guidelines and NAM was obtained from Santa Cruz Biotechnology (Dallas, TX, USA) which was dissolved in fresh 1ml sterile physiologic saline by agitating with high speed and was kept in warm water (30–32 °C) for each dose to avoid precipitation before administration. All antibodies (primary and secondary) used in the present study are shown in (Table 1).

### 2.2. Animal Grouping and Drug Treatment

**Figure 1:**
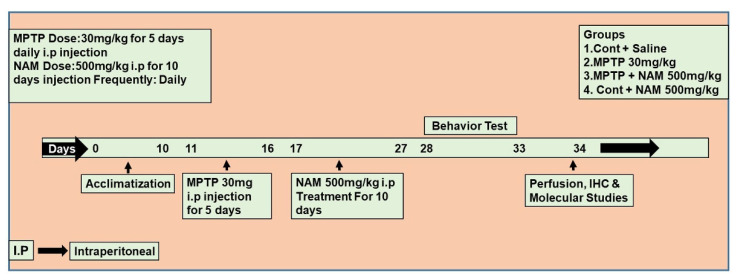
Schematic diagram of the experimental design. Treatment duration and behavioral analysis of the experimental animals.

Wild-type mice (C57BL/6N, eight weeks old, average body weight 25–30 g) were obtained from Samtako Bio labs (Ulsan, Korea). All mice were housed at room temperature under 12 h light/dark cycle and provided food and water. All mice were acclimatized for one week previously [24]. The mice were managed according to the Division of Applied Life Sciences, Gyeongsang National University, South Korea, protocol (approval ID: 125). The mice were divided into four groups (*n* = 10 per group) and divided equally for Western blot (*n* = 5 per group) and immunofluorescence (*n* = 5 per group) analyses: (1) saline-treated control mice; (2) MPTP-treated (30 mg/kg i.p. for 5 days) mice; (3) MPTP + NAM (500 mg/kg, i.p for 10 days)-treated mice; (4) control + NAM-treated mice. The control group was treated with saline (0.9%), MPTP was administered at 30 mg/kg intraperitoneally (i.p) for 5 days, as seen previously [22,25,26,27,28] and NAM was administered at 500 mg/kg i.p [21] for 10 days (Figure 1).

### 2.3. Animal Behavioral Analysis

Before performing the motor task, all the animals were acclimatized for ten days, provided with food and water [29], and were trained in a separate house room before the trials were started at 11 AM.

#### 2.3.1. Open Field Test

The open-field apparatus (40 × 40 cm in diameter and 40 cm in height) was divided into 16 equally sized squares as previously performed. Experimental animals were placed in the center of the arena. A 30 min session was initiated, after putting the mouse in a center of box where the mice were allowed to freely explore in the open field apparatus. The total distance travelled and the time spent in the center were recorded with a SMART video tracking system (Panlab, Holliston, MA, USA) [29,30].

#### 2.3.2. Pole Test

The pole test apparatus consisted of a wood pole (10 mm in diameter and 40 cm in height) [29,31]. The mice were placed on the top of the wood pole with the head in the face-up position and the time taken to reach the floor was measured. The test was repeated three times for 3 trials each carrying 10 min. The behavioral changes were analyzed according to the mean of three descending times.

#### 2.3.3. Wire Hang Test

The mice were placed on the wire, which is mounted horizontally 20 cm above the ground surface. The mice were placed which grabbed the wire with their hands and fore paws [29]. The latency to when the animal falls was recorded. The experimental procedures were repeated 10 times, and the average values were evaluated. The mice were allowed to rest between the trials.

### 2.4. Protein Extraction

After behavior analysis, the mice were anesthetized with ketamine/xylazine and then euthanized. The whole brain tissues were collected and put on a bed of wet ice to chill the mice brain matrix in order to separate the SN and striatum parts of the brain. Following coronal sections, the SN [32] and striatum parts were separated from the brain. The tissues were then separately homogenized in Pro-Prep TM protein extraction solution according to the provided guidelines (iNtRON Biotechnology, Sungnam, Republic of Korea). The samples were centrifuged at 13,000× *g* rpm at 4 °C for 25 min. The supernatants were collected and stored at −70 °C for further analyses and assays.

### 2.5. Western Blot Analysis

Western blot was analyzed as described previously [33,34,35]. Briefly, the protein concentrations were evaluated with a Bio-Rad protein assay kit (Bio-Rad Laboratories, Hercules, CA, USA). Equal amounts of proteins (20 μg) were used for electrophoresed on 10–20% sodium dodecyl sulfate-polyacrylamide gels and transferred to polyvinylidene fluoride (PVDF) membrane (Merck Millipore, St. Louis, MA, USA). Next, membrane was blocked in 5% (*w/v*) skim milk, and then incubated with the primary antibodies (1:1000 dilution) overnight at 4 °C. After incubation with primary antibodies, the membrane was washed and treated with HRP-conjugated anti-rabbit (W401B) and anti-mouse (W402B) Promega (Madison, WI, USA) secondary antibodies. By using a detecting reagents chemiluminescence (ECL), the bands were visualized according to the manufacturer instructions (ATTO, Tokyo, Japan). The optical densities of the bands were examined by using ImageJ software (NIH, Maryland MA, USA).

### 2.6. Lipid Peroxidation (LPO) Assay

For the assessment of oxidative stress, LPO was performed as described previously [36,37]. Briefly, a marker of LPO free malondialdehyde (MDA) was calculated in the tissue homogenate of the striatum and SNpc parts using a lipid peroxidation (MDA) colorimetric/fluorometric assay kit (Bio Vision, Milpitas, CA, USA, Cat. #K739-100), followed by the manufacturer’s protocol.

### 2.7. Reactive Oxygen Species Assay (ROS)

ROS assays was performed as described previously [38,39,40]. Briefly, the brain homogenates of the striatum and SNpc of the different groups were diluted with ice-cold Lock’s buffer at a 1:20 ratio to produce the final concentration of 2.5 mg tissue/500 μL. The reaction mixture containing Lock’s buffer (1 mL, pH ± 7.4), 0.2 mL of homogenate, and 10 mL of 2′-7′dichlorofluorescin diacetate (DCFH-DA) (5 mM) was then incubated at room temperature for 15 min to convert DCFH-DA to the fluorescent product DCF. The conversion of DCFH-DA to DCF was assessed by using a spectrofluorometer (Promega Biosciences USA) with excitation at 484 nm and emission at 530 nm. For background fluorescence (conversion of DCFH-DA in the absence of homogenate), we evaluated parallel blanks. The values were expressed as DCF formed pmol/amount of protein in mg.

### 2.8. Cresyl Violet (Nissl) Staining

For the evaluation of the histological study and degree of neuronal cell death, we performed cresyl violet (Nissl) staining [20,41]. Slides containing 14 µm tissue sections were washed twice for 15 min in 0.01 M PBS. The slides were then stained using 0.1% cresyl violet solution (Sigma-Aldrich, St. Louis, MO, USA) for 15 min. Then, they were dehydrated by 70% ethanol and absolute ethanol. Furthermore, they were treated with xylene to clear tissues and were mounted with DPX mounting medium (Sigma-Aldrich, St. Louis, MO, USA). The sample images were examined by using an Olympus AX70 microscope (Olympus, Tokyo, Japan).

### 2.9. Immunofluorescence Staining

Immunofluorescence staining was performed as described previously [42,43,44]. Briefly, mice were deeply anesthetized and perfused with normal saline solution (0.9%) and 4% cold paraformaldehyde. The brain samples were removed and placed in paraformaldehyde solution for 72 h at 4 °C, followed by immersing in 30% sucrose for the next 72 h. The brain tissues were fixed in optimal cutting temperature (OCT) compound (Sakura Finetek Japan Co., Ltd., Tokyo, Japan), and the midbrain samples comprising the striatum and SNpc were serially cut into 14 μm-thick coronal sections using a cryomicrotome (Leica cryostat CM 3050, Heidelberg, Germany). The slides containing the brain samples were dehydrated at room temperature for 24 h then washed twice with phosphate-buffered saline (PBS) (0.01 mM) for 10 min each. The slides were treated with proteinase K for 5 min, rinsed with PBS (0.01 mM), and blocked with 2% goat normal serum in PBS containing 0.1% Triton X-100 for 50 min. The slides were incubated with primary antibodies overnight at 4 °C including anti-TH (Santa Cruz Biotechnology, Dallas, TX, USA), and α-synuclein (Santa Cruz Biotechnology, Dallas, TX, USA). After incubating with primary antibodies, the slides were incubated with fluorescein isothiocyanate (FITC) or tetramethylrhodamine (TRITC)-labeled secondary antibodies (anti-rabbit or anti-mouse) for 90 min at room temperature. Slides were washed twice with PBS for 5 min each. By using fluorescent mounting medium (Dako 53023), glass coverslips were mounted on slides. Images were taken using a confocal laser-scanning microscope (Olympus FLUOVIEWFV3000 microscope (Olympus, Tokyo, Japan).

### 2.10. Statistical Analysis

The immunoblot bands (X-ray scanned film) and immunofluorescence images (densitometries) were analyzed with ImageJ (version 1.50), and all experimental datasets were statistically assessed by GraphPad Prism 6 software. One-way ANOVA (analysis of variance) was selected based on the Shapiro–Wilk normality test. Results were presented as the mean ± standard deviation (SD). Statistical differences presented as * *p* ≤ 0:05 (for the control group) and ^#^ *p* ≤ 0:05 (for the NAM-treated group) were considered significant when compared both to the MPTP toxic group. Statistical analysis is conferred in the figure legends.

## 3. Results

### 3.1. Effects of NAM on Motor Dysfunctions in the MPTP-Induced PD Model

**Figure 2:**
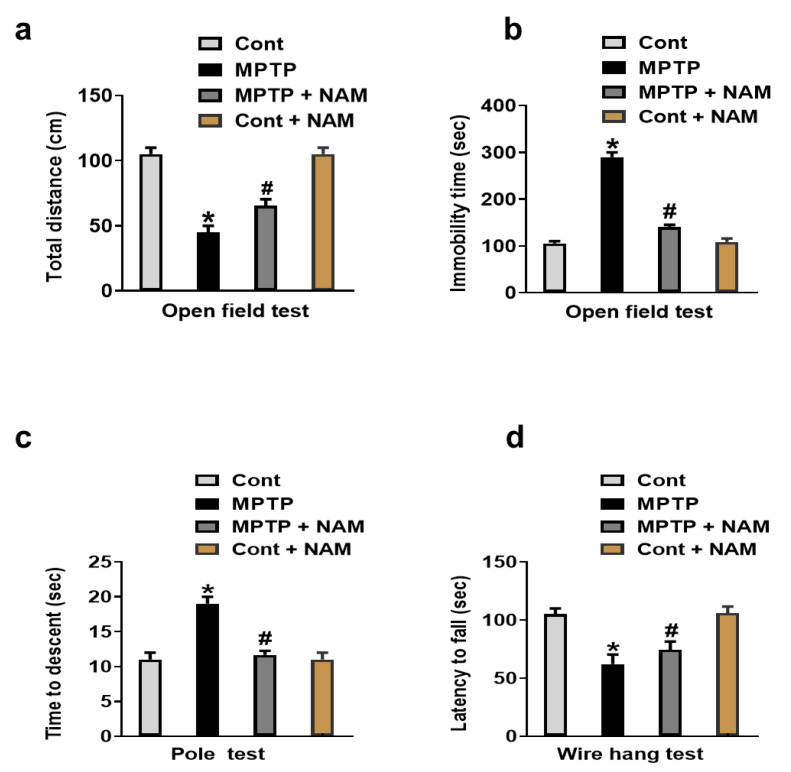
Nicotinamide (NAM) abolished motor dysfunction in MPTP-induced PD model. (**a**,**b**) Quantitative analysis of the total distance covered and immobility time by the mice in the open field box; (**c**) Representative histogram of the pole test results; (**d**) Histogram showing the results of the wire hang test. * Significantly different from the control group; ^#^ Significantly different from MPTP-treated mice. Significance. * *p* < 0.05; ^#^ *p* < 0.05.

Motor dysfunctions and behavior alterations are reported in the MPTP-induced PD model [45]. Therefore, we evaluated the effects of NAM on motor dysfunction and behavior changed through open field test, pole test, and wire hang test. Our results showed reduced total distance and increased immobility time in MPTP-treated mice as compared to the saline-treated control, while treatment with NAM significantly reversed these effects (Figure 2a,b). The pole test showed the descending time was significantly increased in the MPTP induced model, but the treatment with NAM significantly reduced the latency to reach the floor (Figure 2c). Lastly, wire hang test showed reduced hanging time in the MPTP-induced PD mice compared to saline-treated control mice, while mice treated with NAM significantly increased the hanging time (Figure 2d).

### 3.2. NAM Decreased the Expression of α-Synuclein in the MPTP-Induced PD Striatum and SNpc

**Figure 3:**
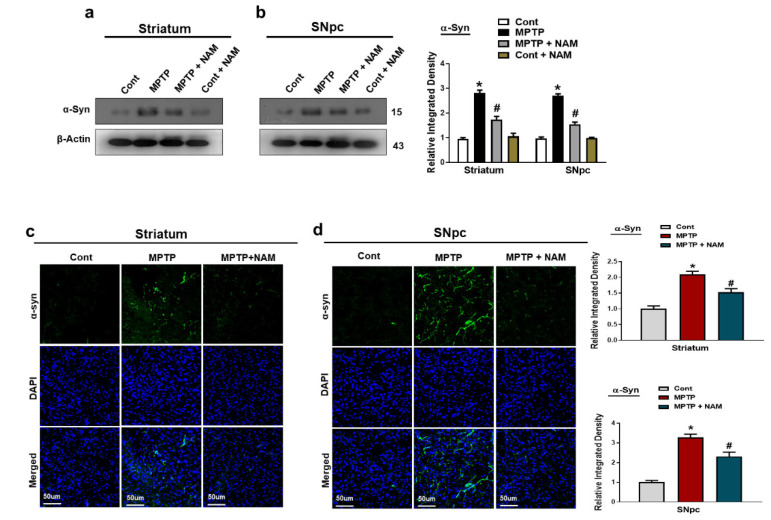
Nicotinamide (NAM) abrogated the α-synuclein level in the Striatum and SNpc of the mouse brain. (**a**,**b**) Western blot analysis of α-synuclein, in the striatum and SNpc of different experimental animals; (**c**,**d**) Immunofluorescence analysis of α-synuclein, (green) and its corresponding histogram and dapi staining (blue) in the striatum and SNpc regions. The densities values are expressed in arbitrary units (AU), magnification 10×, scale bar = 50 µm. The values are expressed as the mean ± SD for indicated proteins (*n* = 10 mice/group), and the number of independent experiments was *n* = 3. * Significantly different from the control group; ^#^ Significantly different from the MPTP-treated group. Significance: * *p* < 0.05; ^#^ *p* < 0.05.

α-synuclein is the main player of the Lewy bodies and the pathogenesis of PD. It has been confirmed that MPTP-induced mice produce α-synuclein in the brain [46]. We also evaluated the effects of NAM on MPTP induced α-synuclein aggregation in striatum and SNpc. Our Western blot results showed increased expression of α-synuclein in MPTP-induced PD mice brain, while NAM treatment significantly decreased the expression (Figure 3a,b). Through immunofluorescence, we further confirmed the beneficial effects of NAM on the MPTP-induced α-synuclein expression in the Striatum and SNpc (Figure 3c,d).

### 3.3. NAM Protected Dopaminergic Neurons by Preserving the TH and DAT Levels in the Striatum and SNpc

**Figure 4:**
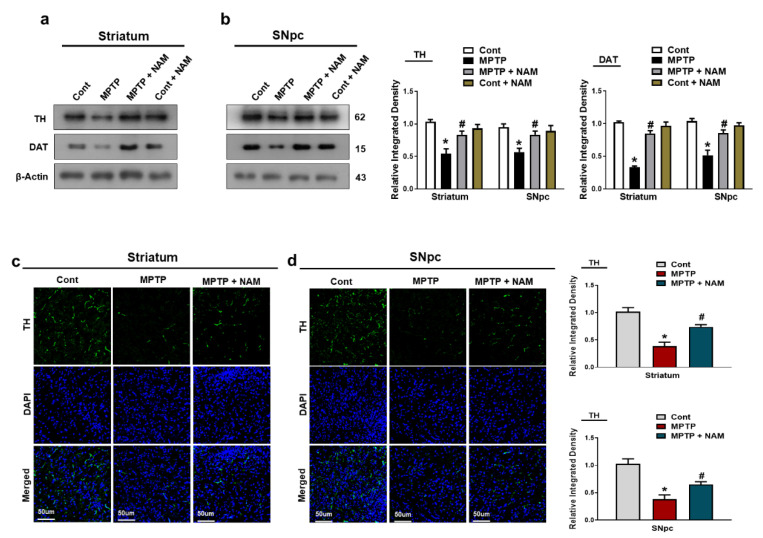
Nicotinamide (NAM) upregulated the TH and DAT level in the striatum and SNpc of the mouse brain. (**a**,**b**) Images of the scanned Western blot results and their representative histograms showing expression of TH and DAT. β-actin was used as a loading control; (**c**,**d**) Immunofluorescence analysis of TH, (green) and its corresponding histogram and dapi staining (blue) in the striatum and SNpc regions. The densities values are expressed in arbitrary units (AU), magnification 10×, scale bar = 50 µm. The values are expressed as the mean ± SD for indicated proteins (*n* = 10 mice/group), and the number of independent experiments was *n* = 3. * Significantly different from the control group, ^#^ Significantly different from the MPTP-treated group. Significance: * *p* < 0.05; ^#^ *p* < 0.05.

TH and DAT are key regulators of dopamine neurotransmission and the loss of these proteins are involved in the pathophysiology of PD [47]. Therefore, we examined the effects of NAM on TH and DAT expressions on MPTP-induced mice. Our Western blot results showed significantly decreased expression of TH and DAT levels in the striatum and the SNpc of MPTP-induced mice compared to saline-treated control mice. Interestingly, NAM treatment significantly increased TH and DAT expressions (Figure 4a,b). Similarly, immunofluorescence analysis showed decreased expression of TH in the SNpc of MPTP-induced mice compared to control mice, while treatment with NAM increased the immunoreactivity of TH in the Striatum and SNpc of MPTP model (Figure 4c,d).

### 3.4. NAM Protected the Striatum and SNpc against MPTP-Induced Oxidative Stress

**Figure 5:**
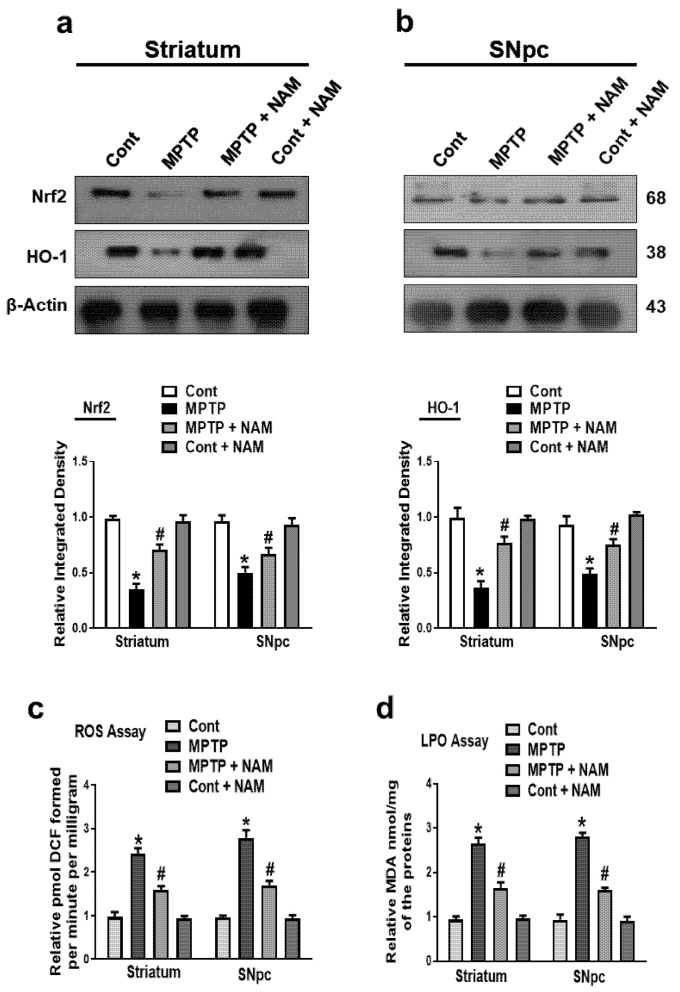
Nicotinamide (NAM) ameliorating oxidative stress and regulated the expression of the Nrf2 and HO-1 pathway in the Striatum and SNpc of mice. (**a**,**b**) Images of the scanned immunoblot results of Nrf2 and HO-1 and their graphical representation in the striatum and SNpc of the different experimental groups. β-actin was used as a loading control; (**c**) Graphical representation of the reactive oxygen species (ROS) level in the striatum and SNpc of the different experimental groups; (**d**) Graphical representation of the results of the lipid peroxidation (LPO) assay in the different experimental groups. The values are expressed as the mean ± SD for indicated proteins (*n* = 10 mice/group), and the number of independent experiments was *n* = 3. * Significantly different from the control group, ^#^ Significantly different from the MPTP-treated group. Significance: * *p* < 0.05; ^#^ *p* < 0.05.

Administration of MPTP to the rodents increased the ROS and oxidative stress [48]. The genes involved in antioxidative stress pathways were also downregulated [49]. We evaluated the effects of NAM on the Nrf2 and HO-1 in the brain of PD mice. Our Western blot results showed that MPTP reduced the expression of Nrf2 and HO-1 in the striatum and SNpc compared to the control mice, while the expression levels were increased by NAM treatment (Figure 5a,b). Additionally, we also performed the ROS and LPO assays in the striatum and SNpc. We found that ROS and LPO levels were significantly elevated in the striatum and SNpc of MPTP-induced mice compared to saline-treated controls. However, ROS and LPO levels were significantly decreased in the striatum and SNpc of NAM-treated MPTP mice (Figure 5c,d).

### 3.5. NAM Ameliorated MPTP-Induced Inflammatory Markers in the Striatum and SNpc

**Figure 6:**
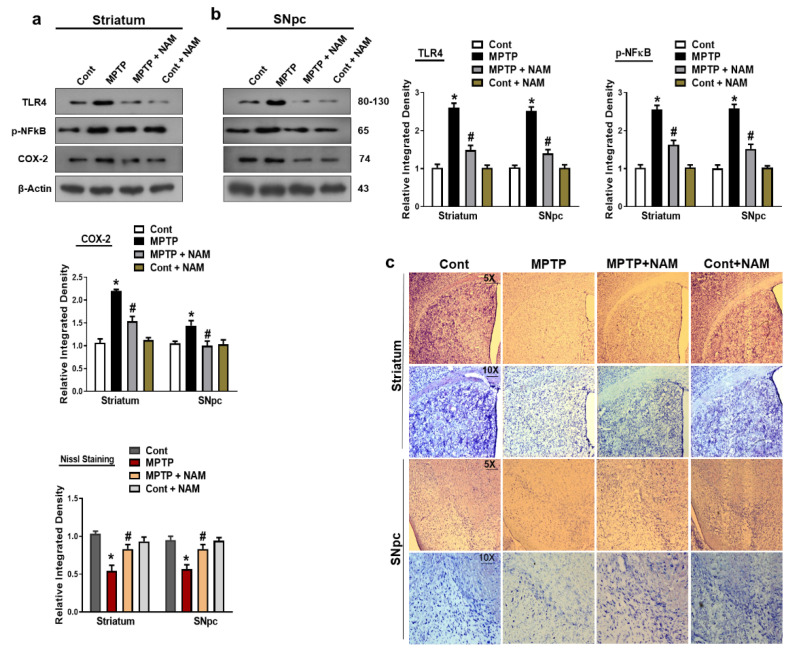
Nicotinamide (NAM) regulated the expression of inflammatory markers. (**a**,**b**) Images of the scanned immunoblot results and their graphical representation of the expression Toll-like receptor 4 (TLR-4), phosphorylated nuclear factor-κB (p-NFκB), and cyclooxygenase-2 (COX-2) in the striatum and SNpc of the different experimental groups. β-actin was used as a loading control; (**c**) Images of the Nissl staining of the striatum and substantia nigra of different experimental groups captured with different magnifications (5× and 10×), respectively. The values are expressed as the mean ± SD for indicated proteins (*n* = 10 mice/group), and the number of independent experiments was *n* = 3. * Significantly different from the control group, ^#^ Significantly different from the MPTP-treated group. Significance: * *p* < 0.05; ^#^ *p* < 0.05.

It has been reported that MPTP increased the expression of inflammatory cytokines in the brain [50]. Therefore, we examined the inflammatory markers Toll-like receptor 4 (TLR4), phosphorylated nuclear factor κB (p-NFκB), and cyclooxygenase-2 (COX-2). The results showed increased expression of these inflammatory markers in the striatum and SNpc of MPTP-induced model compared to controls. Additionally, NAM treatments significantly decreased the expression of these inflammatory markers (Figure 6a,b). The effects of NAM on neuronal degeneration were further analyzed by Cresyl Violet (Nissl) staining, which confirmed that NAM increased the number of Nissl-stained striatum and substantia nigra neurons in MPTP-treated mice (Figure 6c).

## 4. Discussion

The present study investigated the effect of NAM in MPTP-induced mice model for PD. The study has shown that NAM treatment significantly protected neuronal loss and improved the motor functions. Furthermore, NAM treatment abrogated oxidative stress and neuroinflammation in the striatum and SNpc of MPTP-induced mice models compared to controls. Overall, this study has shown NAM treatment exhibited neuroprotective effects in PD pathophysiology and showed potential role in PD treatment.

It is reported that MPTP neurotoxicity brings dopaminergic neurodegeneration and many features of PD such as motor dysfunctions and dopaminergic neurodegeneration [26,51]. Natural drug substances could be potential therapeutic agents for PD and related symptoms since it contains anti-inflammatory and anti-oxidative properties [52,53]. NAM is one of the natural vitamin and essential nutrients that has shown various health benefits across various diseases such as melanoma, chronic kidney diseases [54,55], diabetes mellitus [56], and lungs injuries [57]. NAM also showed neuroprotective properties against Alzheimer’s disease [58], Huntington’s disease [59], hypoxic ischemia [19], traumatic brain injuries, headache, and other psychiatric disorders [60]. We further explored the neuroprotective properties of NAM against MPTP-induced motor dysfunction, oxidative stress neuroinflammation and neurodegeneration. Previous findings suggested that MPTP-treated mice showed reduced motor activities [61]. We also analyzed the effects of NAM on motor activities by conducting the behavior assays, open field test, pole test and wire hang test. Our results showed that NAM treatment improved the motor functions in MPTP-induced PD mice (Figure 2a–d).

Previous findings showed that MPTP treatment significantly increased the expression of α-synuclein in the brain of experimental animals [22,62]. On the other hand, vitamins have been well studied on their abilities to improved PD symptoms [63,64]. In accordance with previous findings, our results also indicated that MPTP injected mice showed increased expression of α-synuclein in the striatum and SNpc. Interestingly, NAM administration significantly decreased the expression of α-synuclein in the brain of PD mice (Figure 3a–c). TH is a rate limiting enzyme and involved in the synthesis, storage and release of dopamine [65]. Similarly, DAT is involved in the recycling of dopamine. TH and DAT plays an important role in the regulation of dopamine in the brain [66]. In PD, dysregulation of TH and DAT occurs in the striatum and SNpc [67]. Our findings also indicated that treatment of MPTP decreased the level of TH, and DAT, while NAM restored the expression of TH, and DAT, in the brain of PD mice (Figure 4a–c).

Accumulation of α-synuclein in the brain activates the production of ROS and oxidative stress, which plays an important role in the degeneration of dopaminergic neurons in PD [68,69]. The antioxidant defense system consists of networks of pathways which alert in response of oxidative stress. Nrf2 is an important sensor of oxidative stress and plays a main role in protecting the cells from excessive oxidative stress [36,70,71]. Nrf2 also regulates the expression several antioxidants defense genes through several mechanism, including HO-1, which removed the toxic heme, carbon monoxide, and iron ions. Additionally, HO-1 protects the cells against ROS and oxidative stress [72,73]. Furthermore, the natural bioactive supplements like vitamins play an important role against oxidative stress and neurodegeneration [74]. According to our findings, NAM treatment significantly potentiated Nrf2 nuclear translocation and upregulated HO-1 expression levels in the MPTP-treated mice, (Figure 5a,b) suggesting the possible antioxidant effect of NAM against MPTP-induced neurodegeneration. Further, we examined ROS and LPO in the mice brains and showed that NAM treatment significantly reduced the expression of ROS and LPO in the striatum and SNpc of the experimental animals (Figure 5c,d).

Indeed, neuroinflammation in the brain is initiated by a variety of toxins [75], which is a prominent risk factor for PD [76]. In MPTP, animal model activates glial cells and elevated proinflammatory factors have been observed in the striatum and SNpc in the brain, which further initiated neuroinflammation and neurodegeneration [77]. TLR4 is a member of TLRs family, and is a basic candidate for initiating innate immune response [78]. It is reported that TLR4 is a key player in the activation of neuroinflammation and neurodegeneration [79]. The increase TLR4 expression in the brain mediated the NF-κB and further release of pro-inflammatory mediators such as COX-2 [80,81]. Notably, studies are demonstrating that NAM is a potent inhibitor of proinflammatory mediators and cytokines [82,83]. Our findings also showed that inflammatory mediators TLR4, p-NF-Κb, and COX-2 were increased in the striatum and SNpc of the MPTP-induced PD mice brain, while treatment with NAM decreased the expression of inflammatory cytokines (Figure 6a,b). Finally, NAM revealed regulatory effects against, oxidative stress, neuroinflammation, motor and cognitive dysfunctions.

## 5. Conclusions

In conclusion, our study provides considerable evidence that NAM can abrogate α-synuclein-induced oxidative stress, neuroinflammation, and motor dysfunctions. NAM+MPTP treatment maintains the cellular antioxidant system and regulates Nrf2/HO-1 protein level. This shows the therapeutic potential of NAM against α-synuclein -accelerated neurotoxicity, and may also open the door for new therapeutic preclinical research work to be carried out.

## Figures and Tables

**Table 1:** List of antibodies used for Western blot and Immunofluorescence analysis.

Name	Source	Application	Manufacturer	Catalog Number	Concentration
TH	Rabbit	WB/IF	Merck Millipore (Burlington, MA, USA)	AB152	1:1000/1:100
DAT	Rat	WB	Santa Cruz Biotechnology (Dallas, TX, USA)	SC: 32259	1:1000
α-synuclein	Mouse	WB/IF	Santa Cruz Biotechnology (Dallas, TX, USA)	SC: 58480	1:1000/1:100
TLR4	Mouse	WB	Santa Cruz Biotechnology (Dallas, TX, USA)	SC: 293072	1:1000
p-NFκB	Mouse	WB	Santa Cruz Biotechnology (Dallas, TX, USA)	SC: 293072	1:1000
Nrf2	Mouse	WB	Santa Cruz Biotechnology (Dallas, TX, USA)	SC: 365949	1:1000
HO-1	Mouse	WB	Santa Cruz Biotechnology (Dallas, TX, USA)	SC: 136961	1:1000
COX-2	Rabbit	WB	Santa Cruz Biotechnology (Dallas, TX, USA)	SC: 7951	1:1000
β-Actin	Mouse	WB	Santa Cruz Biotechnology (Dallas, TX, USA)	SC: 47778	1:1000

Abbreviations. Western blot (WB), Immunofluorescence (IF), Tyrosine hydroxylase (TH), Dopamine Transporters (DAT), Nuclear factor erythroid 2-related factor 2 (Nrf2), Heme oxygenase-1 (HO-1), Toll-like receptor 4 (TLR-4), Phosphorylated nuclear factor-κB, tumor (p-NFκB), cyclooxygenase-2 (COX-2).

## Data Availability

The authors hereby declare that the data presented in this study will be presented upon request from the corresponding author.
